# Rapid spread of a new West Nile virus lineage 1 associated with increased risk of neuroinvasive disease during a large outbreak in Italy in 2022

**DOI:** 10.1093/jtm/taac125

**Published:** 2022-11-04

**Authors:** Luisa Barzon, Monia Pacenti, Fabrizio Montarsi, Diletta Fornasiero, Federica Gobbo, Erika Quaranta, Isabella Monne, Alice Fusaro, Andrea Volpe, Alessandro Sinigaglia, Silvia Riccetti, Emanuela Dal Molin, Sorsha Satto, Vittoria Lisi, Federico Gobbi, Silvia Galante, Giuseppe Feltrin, Valerio Valeriano, Laura Favero, Francesca Russo, Matteo Mazzucato, Alessio Bortolami, Paolo Mulatti, Calogero Terregino, Gioia Capelli

**Affiliations:** Department of Molecular Medicine, University of Padova, via A Gabelli 63, Padova 35121, Italy; Microbiology and Virology Unit, Padova University Hospital, via Giustiniani 2, Padova 35128, Italy; Microbiology and Virology Unit, Padova University Hospital, via Giustiniani 2, Padova 35128, Italy; Istituto Zooprofilattico Sperimentale delle Venezie, Viale dell’Università, 10 - Legnaro (PD), Italy; Istituto Zooprofilattico Sperimentale delle Venezie, Viale dell’Università, 10 - Legnaro (PD), Italy; Istituto Zooprofilattico Sperimentale delle Venezie, Viale dell’Università, 10 - Legnaro (PD), Italy; Istituto Zooprofilattico Sperimentale delle Venezie, Viale dell’Università, 10 - Legnaro (PD), Italy; Istituto Zooprofilattico Sperimentale delle Venezie, Viale dell’Università, 10 - Legnaro (PD), Italy; Istituto Zooprofilattico Sperimentale delle Venezie, Viale dell’Università, 10 - Legnaro (PD), Italy; Department of Molecular Medicine, University of Padova, via A Gabelli 63, Padova 35121, Italy; Department of Molecular Medicine, University of Padova, via A Gabelli 63, Padova 35121, Italy; Department of Molecular Medicine, University of Padova, via A Gabelli 63, Padova 35121, Italy; Department of Molecular Medicine, University of Padova, via A Gabelli 63, Padova 35121, Italy; Microbiology and Virology Unit, Padova University Hospital, via Giustiniani 2, Padova 35128, Italy; Microbiology and Virology Unit, Padova University Hospital, via Giustiniani 2, Padova 35128, Italy; Department of Infectious-Tropical Diseases and Microbiology, IRCCS Sacro Cuore Don Calabria Hospital, Negrar di Valpolicella, Italy; UOC Medicina Trasfusionale, Sede di Camposampiero, Azienda ULSS6 Euganea, via Cosma, 1 - Camposampiero (PD), Italy; Regional Transplant Centre, Padova University Hospital, Via Giustiniani 2, Padova 35128, Italy; Dipartimento di Prevenzione, Azienda ULSS6 Euganea, Servizio di Igiene e Sanità Pubblica, UOSD Epidemiologia e Ambiente, Via Ospedale Civile, 22, Padova 35100, Italy; Direzione Prevenzione, Sicurezza Alimentare, Veterinaria, Regione Veneto, Dorsoduro, 3493 - Rio Novo, Venezia 30123, Italy; Direzione Prevenzione, Sicurezza Alimentare, Veterinaria, Regione Veneto, Dorsoduro, 3493 - Rio Novo, Venezia 30123, Italy; Istituto Zooprofilattico Sperimentale delle Venezie, Viale dell’Università, 10 - Legnaro (PD), Italy; Istituto Zooprofilattico Sperimentale delle Venezie, Viale dell’Università, 10 - Legnaro (PD), Italy; Istituto Zooprofilattico Sperimentale delle Venezie, Viale dell’Università, 10 - Legnaro (PD), Italy; Istituto Zooprofilattico Sperimentale delle Venezie, Viale dell’Università, 10 - Legnaro (PD), Italy; Istituto Zooprofilattico Sperimentale delle Venezie, Viale dell’Università, 10 - Legnaro (PD), Italy

**Keywords:** West Nile virus, lineage, fever, neuroinvasive disease, mosquito, bird

## Abstract

**Background:**

A new strain of West Nile virus lineage 1 (WNV-1) emerged in the Veneto Region, northern Italy, in 2021, 8 years after the last WNV-1 outbreak in Italy. The virus, which co-circulates with West Nile virus lineage 2 (WNV-2), has become endemic in the region, where, in 2022, most human cases of neuroinvasive disease (WNND) reported in Europe have occurred.

**Methods:**

Comparative analysis of the epidemiology and clinical presentation of WNV-1 and WNV-2 infection in humans, as well as the temporal and geographic distribution of WNV-1 and WNV-2 among wild birds and *Culex pipiens* mosquitoes in Veneto, from 16 May to 21 August 2022, to determine if the high number of WNND cases was associated with WNV-1.

**Results:**

222 human cases of WNV infection were confirmed by molecular testing, including 103 with West Nile fever (WNF) and 119 with WNND. The WNV lineage was determined in 201 (90.5%) cases, comprising 138 WNV-1 and 63 WNV-2 infections. In addition, 35 blood donors tested positive, including 30 in whom WNV lineage was determined (13 WNV-1 and 17 WNV-2). Comparative analysis of the distribution of WNV-1 and WNV-2 infections among WNND cases, WNF cases and WNV-positive blood donors showed that WNND patients were more likely to have WNV-1 infection than blood donors (odds ratio 3.44; 95% confidence interval: 1.54–8.24; *P* = 0.0043). As observed in humans, in wild birds, WNV-1 had a higher infection rate (IR) and showed a more rapid expansion than WNV-2. At variance, the distribution of the two lineages was more even in mosquitoes, but with a trend of rapid increase of WNV-1 IR over WNV-2.

**Conclusions:**

Comparative analysis of WNV-1 vs WNV-2 infection in humans, wild birds and mosquitos showed a rapid expansion of WNV-1 and suggested that WNV-1-infected patients might have an increased risk to develop severe disease.

## Introduction

West Nile virus (WNV) is a neurotropic flavivirus transmitted among wild birds by *Culex* spp. mosquitoes in the enzootic cycle. Humans and other mammals may be incidentally infected through mosquito bites, but they represent dead-end hosts. Infection in humans is asymptomatic in most cases, while ~20% of infected people develop flu-like illness [West Nile fever (WNF)] and <1%, especially elderly and immunocompromised individuals, present with neuroinvasive disease (WNND).^1^

The virus, which originated from sub-Saharan Africa, has spread globally and has become endemic in several countries in Europe, the Americas, Asia and Oceania.^2^ Phylogenetic analysis identified nine evolutionary lineages of which West Nile virus lineage 1 (WNV-1) and West Nile virus lineage 2 (WNV-2) have been associated with disease in humans.^3^ Different strains of WNV-1 circulated in Europe and in the Mediterranean basin, causing human outbreaks since at least the late 1950s, while WNV-2 was first detected in 2004 in Hungary^4^ and then spread to several central and southern European countries, causing most infections in humans.^5^^,^^6^

During the last three decades, different strains of both WNV-1 and WNV-2 have been introduced in Italy, one of the most affected countries in Europe.^7^ WNV-1 was first detected in Italy in 1998 during an equine outbreak; after 10 years, other WNV-1 strains of the Western Mediterranean clade caused a large human and equine outbreak in the Po river basin area in northern Italy in 2008–09 and 2011–13 and in southern Italy and Sardinia in 2010–11.^7^ WNV-2 of the Central and Southern European clade was first detected in Italy in 2011^8^ and replaced WNV-1 in northern Italy since 2013, causing large human outbreaks in 2013–14^9^ and 2018–19.^10^^,^^11^ Surveillance data showed that the Central and Southern European clade of WNV-2 has become predominant in Europe, where its geographic range is expanding. During the last decade, this clade represented the exclusively detected WNV in several European countries, including Austria, Bulgaria, Croatia, Germany, Greece, Hungary, the Netherlands and Romania,^12^ while WNV-1 was sporadically detected in birds and mosquitoes in France (2015 and 2018) and Italy (2015–17 and 2020)^13^^,^^14^ and during the human outbreak that occurred in Spain in 2020.^15^

These epidemiological data show how WNV can spread at short and long distances among European and Mediterranean countries, where the virus can not only establish, overwinter and become endemic but also extinguish. Monitoring WNV epidemiology and the emergence of new viral strains is crucial to understand the WNV biology and ecology and to timely trigger public health response measures. This is particularly relevant in the context of climate change, which has a dramatic impact on mosquito-borne infections by influencing the distribution of amplifying hosts and vector populations.

We recently reported a new strain of WNV-1, which was first detected in the Veneto Region, northern Italy, in 2021, 8 years after the last outbreak of WNV-1 in Italy.^16^ The virus, which co-circulates with WNV-2, is rapidly expanding in the region, where, in 2022, most human cases of WNND and associated mortality reported in Europe have occurred.^17^ In this study, we applied a ‘One Health’ approach to evaluate the epidemic potential and virulence of the new WNV-1 strain. In particular, we compared the epidemiology and clinical presentation of WNV-1 vs WNV-2 infection in humans as well as the rate of WNV-1 vs WNV-2 infections in wild birds and *Culex pipiens* mosquito. This analysis showed a rapid expansion of WNV-1 and suggested that WNV-1-infected individuals might have an increased risk to develop WNND.

## Materials and Methods

### Ethics statement

The cases reported in this study were investigated with routine procedures according to the national surveillance plan for arbovirus infections. Therefore, no approval was required from the ethics committee. Written informed consent was obtained from the study subjects.

### Human surveillance

In Italy, WNV surveillance is carried on according to the National Plan for Prevention, Surveillance and Response to Arboviruses 2020–25,^16^^,^^18^^,^^19^ which integrates human, veterinary and entomological surveillance in a ‘One Health’ approach. According to the plan, WNV surveillance in humans is conducted all year round in the entire national territory and is enhanced during the transmission season, from May to November, in areas classified as at risk, like Veneto Region provinces (areas at risk are NUTS3 with ongoing WNV activity or with evidence of WNV activity in at least 1 of the previous 5 years). Human surveillance targets individuals with neurological symptoms or with febrile illness. Confirmed WNV cases are defined as individuals (with or without symptoms) presenting with at least one of the following laboratory criteria: virus isolation from serum, urine and/or cerebrospinal fluid (CSF); detection of viral RNA in blood, urine and/or CSF; detection of a specific IgM antibody response in CSF; high IgM antibody titre and detection of IgG antibodies in serum and confirmation by neutralization assays. Probable WNV cases are defined as individuals with fever or neurological symptoms and IgM antibodies against WNV detected in serum. The period considered in this study ranged from Week 20 (16–22 May 2022) to Week 33 (15–21 August 2022). Data were aggregated on a biweekly basis and by province to allow comparisons with the observations made on wild bird and mosquito data.

### Surveillance of wild birds

Surveillance of WNV was based on testing wild birds found dead in the regional territory (passive surveillance) and delivered to the regional centres for wild animals and also on birds samples related to the active surveillance in target species (mainly corvids, e.g. magpie, *Pica pica*; carrion crow, *Corvus corone cornix* and Eurasian jay, *Garrulus glandarius*) according to the National Plan for Prevention, Surveillance and Response to Arboviruses 2020–25.^18^ The period considered in this study ranged from Week 20 (16–22 May) to Week 32 (8–14 August). Data were aggregated on a biweekly basis and by province.

### Entomological surveillance

Entomological surveillance was carried out all over the flatlands of Veneto Region (<300 m above the sea level) from May to October using 55 CDC-like traps (Centers for Disease Control and Prevention-like trap; Italian Mosquito Trap IMT®; PeP, Cantu, Italy) baited with CO_2_. The traps run for one night every 2 weeks. Mosquitoes were pooled according to species and tested for WNV infection. Pooled female specimens of *Cx. pipiens*, *Culex modestus*, *Aedes albopictus* and *Ochlerotatus caspius* species were tested for WNV. Only *Cx. pipiens*, which represented >97% of WNV-positive mosquito pools, in agreement with previous reports,^20^ were considered for the analyses. The period ranged from the day of the first capture of *Cx. pipiens* on 7 May–11 August. Data were aggregated on a biweekly basis and by province.

### Laboratory methods

Laboratory investigation of suspected WNV infections in humans was carried out as previously described.^21^ Briefly, for viral RNA detection, total nucleic acids were purified from 200 μl of whole blood, plasma, urine or CSF by using a MagNA Pure 96 System (Roche Diagnostics, Basel, Switzerland) and were amplified by two in-house real-time RT-PCR methods, which allowed the discrimination between WNV-1 and WNV-2.^22^^,^^23^ Real-time RT-PCR assays were carried on using the one-step real-time RT-PCR kit (Thermo Fisher Scientific, Waltham, Massachusetts, USA) and run on ABI 7900HT Sequence Detection Systems (Thermo Fisher Scientific). In addition, the cobas® WNV Test on a cobas® 6800 System (Roche Diagnostics) was used to detect WNV RNA in 1000 μl of plasma samples. This test is highly sensitive but cannot discriminate between WNV-1 and WNV-2. Testing for other vector-borne viruses (Usutu virus, USUV; Toscana virus, tick-borne encephalitis virus, dengue virus, Zika virus and chikungunya virus) was included in the differential diagnosis, as reported.^19^

The presence of WNV IgM and IgG antibodies in serum and CSF was determined by a commercial ELISA (WNV IgM capture DxSelect e IgG DxSelect, Focus Diagnostics, CA, USA). Serum samples with positive results were further tested for confirmation by plaque reduction neutralization test against WNV and microneutralization titre assay against the antigenically related USUV, as reported.^25^

For entomological surveillance, mosquitoes were morphologically identified, pooled (100 specimens maximum) and screened for flaviviruses by using an in-house developed one-step SYBR green-based real-time RT-PCR assay, as previously described.^26^ All Flavivirus RNA-positive mosquito pools were directly sequenced to differentiate WNV, USUV or other flaviviruses.

For wild bird surveillance, a pool of organs (brain, spleen, heart and kidney) was collected from each bird and was submitted for RNA extraction by QIAsymphony DSP Virus/Pathogen Midi kit (QIAGEN) and multiplex real-time RT-PCR amplification of WNV-1, WNV-2 and USUV.^27^ Amplification reaction was assembled with the QuantiTect Probe RT-PCR kit (QIAGEN) using CFX 96 Deep well Real-Time PCR System, C1000 Touch (Biorad, Hercules, CA, USA).

Whole-genome sequencing of WNV-1 was performed by amplicon sequencing directly on viral RNA isolated from biological specimens. Briefly, total RNA was extracted from plasma samples and urine samples collected from humans, from mosquito pools and from organs of dead birds using QIAamp Viral RNA mini kit (QIAGEN) following the manufacturer’s instructions. SuperScript™ III One-Step RT-PCR System with Platinum™ Taq High Fidelity DNA Polymerase (Invitrogen) was used to amplify whole WNV-1 genome using the primer sets reported in Supplementary Table S1. Sequencing was performed using Illumina MiSeq (2 × 300 bp PE). Complete genomes were generated through a reference-based approach. High-quality reads were aligned against a reference genome using BWA v0.7.12,^28^ processing the alignments with Picard-tools v2.1.0^29^ and GATK v3.5.^28^^,^^30^^,^^31^ Single-nucleotide polymorphisms were called using LoFreq v2.1.2.^32^ Sequences were submitted to the GenBank database under the accession numbers OP609791-OP609810 and OP609812-OP609815. Complete genome sequences were aligned together with a dataset of WNV-1 sequences available in GenBank using default parameters in MAFFT version 7.^33^^,^^34^ The maximum-likelihood phylogenetic tree was constructed using IQ-TREE and GTR + F + I + G4 model of nucleotide substitution) with 1000 ultrafast bootstrap replicates.

### Statistical analysis

Comparisons among groups were done by Pearson’s *χ*^2^ test, Student’s *t* test, Fisher’s exact test and Wilcoxon-Mann–Whitney test, as appropriate. Results were considered to be statistically significant with a *P* value ≤ 0.05.

Descriptive exploratory graphs and estimation of the virus infection rate (IR) in birds and mosquitoes were performed using R version 4.2.1^24^ and the packages ggplot2^35^ and PooledInfRate.^36^ Specifically, the bias-reduced maximum likelihood estimate (MLE) based on Firth’s correction was used for the estimation of the mosquito IR and the skewness-corrected score interval for the calculus of the relative 95% confidence intervals (CIs). The MLE method used to estimate the IR does not rely on the assumption that a positive pool contains only one infected mosquito,^37^ as it is assumed in the classical minimum infection rate calculation and which might be not completely correct and lead to biased interpretations.

## Results

### Comparative analysis of WNV-1 and WNV-2 infection in humans

**Figure 1:**
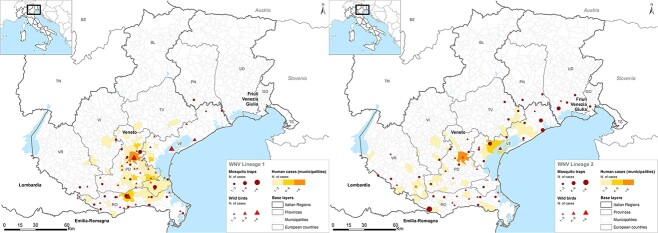
Geographical distribution of WNV-1 (right panel) and WNV-2 (left panel) infections in humans, birds and mosquitoes in the Veneto Region as of 12 August 2022.

**Figure 2:**
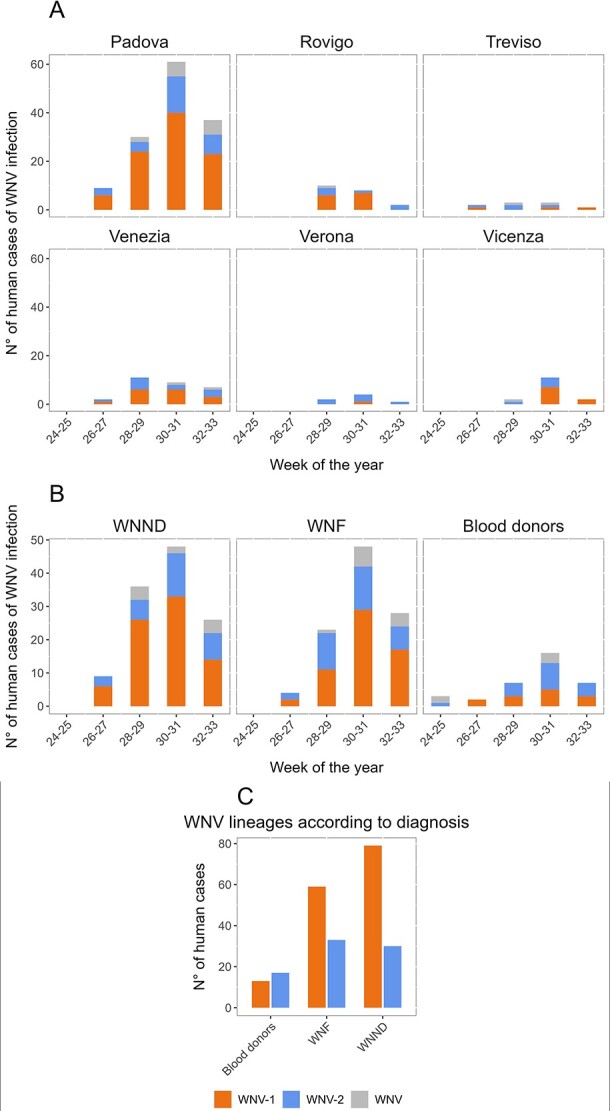
Temporal distribution of human cases of WNV infection according to WNV lineage, week (aggregated on a biweekly basis) of symptom onset and (A) provinces of the Veneto Region and (B) diagnosis (WNND, WNF, blood donors); (C) distribution of human cases of WNV infection according to WNV lineage and diagnosis; *P* = 0.0043, Chi-square test for trend).

As of 21 August 2022 (Week 33), 222 human cases of WNV infection were confirmed by molecular testing at the reference laboratory of Veneto Region, including 103 cases with fever (WNF) and 119 with neuroinvasive disease (WNND). Of these cases, 201 (90.5%) were classified according to the lineage, comprising 138 WNV-1 and 63 WNV-2 infections (Table 1). The number of WNV infections increased steadily each week from Week 26 in July to Week 31 in August 2022, with a higher proportion of WNV-1 cases than WNV-2 cases in Padova (76%), Rovigo (68%) and Venezia (59%) provinces, while WNV-2 was more frequent in the Verona province (86%; Figures 1 and 2A). Most infections occurred in rural and periurban environments.

**Table 1:** Characteristics of cases of WNV infection by status and lineage

	Patients		Blood donors	
Feature	WNV-1 (*n* = 138)	WNV-2 (*n* = 63)	WNV-1 (*n* = 13)	WNV-2 (*n* = 17)
Female, no. (%)	49 (35.5)	23 (36.5)	2 (15.4)	6 (35.3)
Male, no. (%)	89 (64.5)	40 (63.5)	11 (84.6)	11 (64.7)
Age, median years (IQR)	71 (56–80)	74 (55–82)	51 (43–53)	52 (44–58)
Week of symptom onset, median (minimum)	30 (26)	30 (26)	30 (27)	30 (24)
Days from onset to sampling, median (IQR)	4 (2–7)	5 (2.5–9)	−1 (−1 to 0)	−1 (−1 to 2)
Province				
Padova (PD), no. (%)	94 (68.1)	29 (46.0)	8 (61.5)	5 (29.4)
Venezia (VE), no. (%)	16 (11.6)	13 (20.6)	3 (23.1)	6 (35.3)
Rovigo (RO), no. (%)	13 (9.4)	6 (9.5)	0 (0)	2 (11.8)
Vicenza (VI), no. (%)	10 (7.2)	5 (7.9)	1 (7.7)	0 (0)
Verona (VR), no. (%)	2 (1.4)	6 (9.5)	0 (0)	3 (17.6)
Treviso (TV), no. (%)	3 (2.2)	4 (6.3)	1 (7.7)	1 (5.9)
Symptoms				
Any symptom, no. (%)	138 (100)	63 (100)	5 (38.5)	9 (52.9)
Fever, no. (%)	123 (89.1)	52 (82.5)	0 (0)	3 (17.6)
Athralgia, no. (%)	38 (27.5)	21 (33.3)	1 (7.7)	4 (23.5)
Myalgia, no. (%)	38 (27.5)	21 (33.3)	2 (15.4)	4 (23.5)
Headache, no. (%)	54 (39.1)	36 (57.1)*	1 (7.7)	5 (29.4)
Asthenia, no. (%)	77 (55.8)	30 (47.6)	2 (15.4)	7 (41.2)
Rash, no. (%)	32 (23.2)	10 (15.9)	2 (15.4)	1 (5.9)
Gastrointestinal symptoms, no. (%)	25 (18.1)	10 (15.9)	0 (0)	0 (0)
Neurological symptoms, no. (%)	79 (57.2)	30 (47.6)	0 (0)	0 (0)

^*^WNV-1 vs WNV-2, *P* = 0.02 by Fisher’s exact test.

From 15 June to 21 August 21, ~50 000 blood donations were collected in the Veneto Region and were screened by WNV nucleic acid testing. Overall, 35 blood donors tested positive, including 30 in whom the WNV lineage was determined (13 with WNV-1 and 17 with WNV-2 infection; Table 1). Half of the blood donors developed symptoms after donation. The temporal distribution of the WNV-positive index blood donations is shown in Figure 2B. Most WNV-positive donors were resident in the Padova (40%) and Venezia (31%) provinces. No significant differences between the WNV-1- and WNV-2 positive blood donors were observed concerning age, sex and occurrence of symptoms after donation.

Comparison between patients with WNV-1 infection and those with WNV-2 infection, regarding demographic characteristics and clinical presentation, showed no significant differences, except headache that was more frequent in patients with WNV-2 infection than in those with WNV-1 infection. However, significant differences emerged when patients were compared with WNV-positive blood donors, who can be considered an indicator of the incidence of WNV infection in the general population. In fact, comparative analysis of the distribution of WNV-1 and WNV-2 infections among WNND cases, WNF cases and WNV-positive blood donors showed that patients with WNND were more likely to have WNV-1 infection than blood donors (odds ratio 3.44; 95% CI: 1.54–8.24; *P* = 0.0043 Fisher’s exact test). Trend analysis showed a significant association between WNV-1 infection and the severity of the clinical condition (blood donors, WNF, WNND, *P* = 0.0043 Chi-square test for trend, Figure 2C).

### Comparative analysis of WNV-1 and WNV-2 infection in wild birds

**Figure 3:**
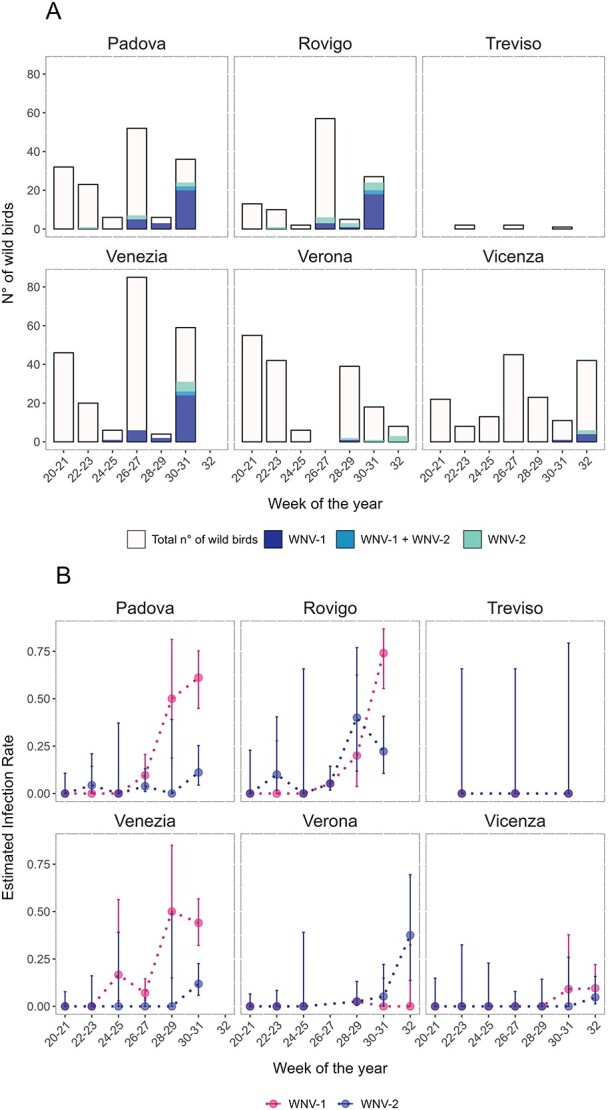
(A) Number of tested and WNV-positive wild birds per province on a biweekly basis; the dashed line indicates the total number of tested animals (right axis); (B) biweekly MLE for WNV-1 and WNV-2 bird infection rate per province; vertical lines represent the 95% CIs.

The first WNV-positive wild birds were found in the Padova and Vicenza provinces at the beginning of June (Week 23). Laboratory analyses confirmed the virus belonged to WNV-2, while WNV-1 was first identified in wild birds sampled during Week 25 in Venezia province. In the following weeks, WNV-1 markedly increased among the positive samples, especially in the Padova, Venezia and Rovigo provinces, reaching a peak in Weeks 30–31 (Figure 3). Six wild birds were found co-infected with both WNV lineages: a common kestrel (*Falco tinnunculus*) and a little owl (*Athene noctua*) in the Padova province, two little owls (*A. noctua*) in Rovigo and a common woodpigeon (*Columba palumbus*) and a Eurasian jay (*G. glandarius*) in the Venezia province. A list of the wild bird species that tested positive for WNV-1 and/or WNV-2 lineages is reported in Supplementary Table S2.

As observed in humans, in wild birds, WNV-1 had higher IR in the Padova, Venezia and Vicenza provinces. In the Verona province, WNV-2 was more present than WNV-1, while in Rovigo, the trend shifted from a higher or equal proportion of WNV-2 vs WNV-1, until the Weeks 28–29, to an inversion in the proportions in Weeks 30–31, with a marked increase in the number of WNV-1 findings (Figure 3).

### Comparative analysis of WNV-1 and WNV-2 infection in mosquitoes

**Figure 4:**
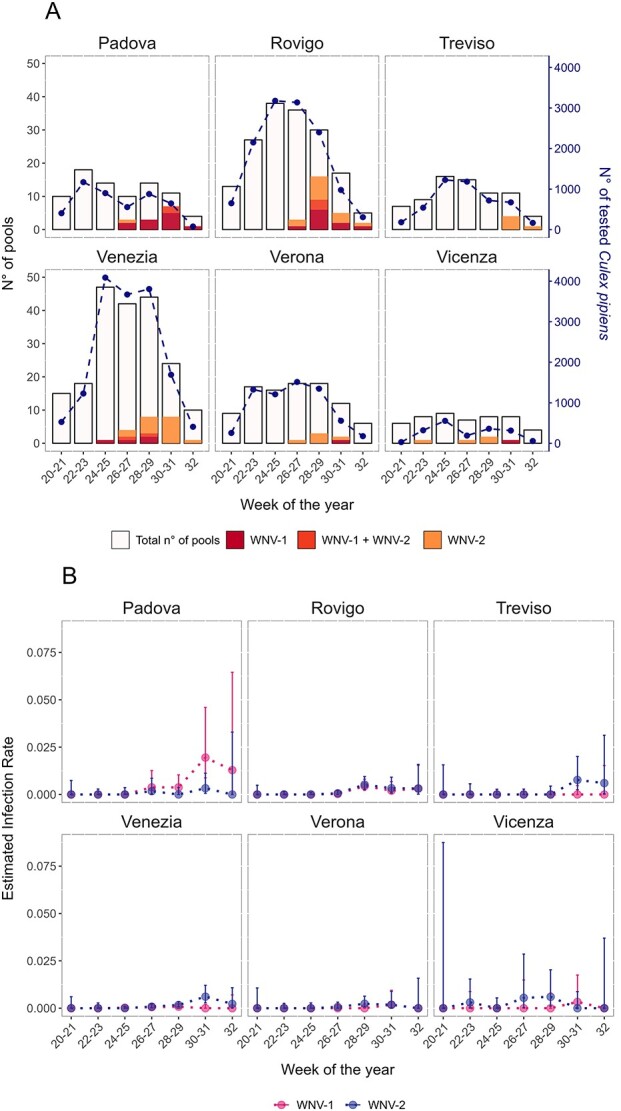
(A) Number of tested and WNV-positive *Cx. pipiens* pools per province on a biweekly basis; the dashed line indicates the total number of tested mosquitoes (right axis); (B) biweekly MLE for WNV-1, and WNV-2 mosquito infection rate per province; vertical lines represent the 95% CIs.

The first WNV-positive *Cx. pipiens* pool, classified as WNV-2, was retrieved in Vicenza province at the beginning of June (Week 23). Two weeks later (Week 25), WNV-1 emerged in mosquitoes collected in the Venezia province (Figure 4). In the following weeks, both lineages spread rapidly in the other provinces of Veneto, with a marked presence in Padova, Venezia and Rovigo. In seven of the investigated pools, both lineages were identified (three in Rovigo and two in Padova and Venezia), indicating overlapping geographical distributions.

Overall, WNV-2 resulted in having a higher IR in five out of six provinces included in the analyses. At variance, in the Padova province, the MLE values for WNV-1 overtook those calculated for WNV-2 starting on Weeks 26–27 (early Summer) and peaked 1 month later, with the highest MLE values calculated since the beginning of the study period (Figure 4).

### Whole-genome sequencing and phylogenetic analysis of WNV-1

In this study, we generated new full WNV-1 genome sequences from samples of nine patients, 13 wild birds and three mosquito pools, which were collected in different areas representative of the regional territory in the period from June to August 2022. Phylogenetic tree analysis of these genomes showed clustering within the Western Mediterranean clade, where they generate a monophyletic group with the WNV-1 genomes detected in 2021 in the Veneto Region (Supplementary Figure S1). Comparison of these genomes showed a similarity of 99.81–100% and 99.76–100% at the nucleotide and protein levels, respectively, with no genetic clustering according to the species or province of collection. In the phylogenetic tree, the highest similarity (98.48% nucleotide and 99.75% amino acid similarity) was observed with the genome of a WNV-1 isolate from a horse with neuroinvasive disease from the Camargue region in Southern France in 2015 (WNV-Akela/France/2015, GenBank MT863559.1). At variance, higher sequence divergence was observed with American WNV-1 strains (~95.33% nucleotide and 99.59% amino acid similarity). Pairwise alignment of the Italian WNV-1 genomes with the closest phylogenetically related WNV-1 strain Akela/France/2015 identified the following amino acid substitutions as present is all Italian genomes but not in the France strain: four conservative amino acid changes in NS2A (R122H) and NS5 proteins (R4K, K44R and K432R) and three non-conservative mutations in NS3 helicase/protease (H244Q and T249P) and NS4B (R15S) protein. Comparison of the Italian WNV-1 genomes with American WNV-1 genomes highlighted the presence of the E-I159V or E-I159A mutations in American strains (but not in Italian genomes), which have been associated with increased viral replication in the brain of mice after intracranial injection,^38^ while the non-conservative mutations NS3-H244Q, NS3-T249P and NS4B-R15S detected in the Italian genomes were also present in the American strains.

### Detection of USUV in humans, horses, wild birds and mosquitoes, Veneto Region, 2022

According to the national integrated surveillance plan for WNV and USUV, we also tested USUV infection in humans, horses, wild birds and mosquitoes. Like WNV, USUV is endemic in the Veneto Region, where it shares the same transmission cycle with WNV.^5^^,^^6^ In the period from 15 May to 21 August 2022, no human cases of USUV infection were identified in the Veneto Region nor were USUV infections identified among wild birds. At variance, the veterinary and entomological surveillance detected USUV RNA in nine wild birds (Supplementary Table S2) and in nine mosquito pools, three of which were also co-infected with WNV-2 and one with both WNV-1 and WNV-2 lineages.

## Discussion

In 2022, Italy is facing a large outbreak of WNV infection and is reporting most of the human cases of infection detected in the EU/EEA countries. This situation is at variance with the large WNV outbreak in 2018, which involved several European countries.^11^ Notably, in 2022, in Italy, most of the human cases of infection, and especially cases of neuroinvasive disease and associated mortality, were detected in the Veneto Region,^17^^,^^39^ where a newly introduced WNV-1 strain was co-circulating with WNV-2.^16^ In this study, we explored the hypothesis that this WNV-1 strain could account for the increased disease burden observed in the region. By applying an integrated analysis of data achieved from human, wild birds and mosquito surveillance, we demonstrated a rapid expansion of WNV-1 in the Padova province, where the virus was first detected in 2021,^16^ and its spread to neighbouring provinces, where it has become the dominant strain. The bird reservoir probably played a key role in viral spread, as suggested by the rapid and marked increase of the proportion of WNV-1-infected wild birds compared with the WNV-2-positive birds. Exceptionally warm spring temperatures and prolonged draught might have anticipated WNV amplification and dispersal among a susceptible bird population.^40^ In addition, the loss of extensive wetland habitats due to the severe scarcity of water may have favoured the aggregation of wild birds, thus increasing the contact rate between multiple wild bird species and vectors. The newly introduced WNV-1 strain might have an advantage over WNV-2, which has been circulating and causing outbreaks in Italy during the last 10 years, with mechanisms that warrants investigation, such as immune escape in vertebrate and invertebrate hosts, increased infectiousness, transmissibility, replication efficiency and/or expansion of the host range.^41^ Likewise, in 2013, the recently introduced WNV-2 rapidly spread in northern Italy, causing a large human epidemic and displacing previously circulating WNV-1 strains.^42^ The extremely high temperatures and drought recorded during the 2022 Spring and Summer seasons^16^ might also have facilitated the evolution and adaptation of the virus to novel ecological conditions.^43^ Considering the current trend of rapid expansion of this WNV-1 strain, it is conceivable that the virus will further expand its geographical range in the future.

The higher activity of WNV-1 than WNV-2 in the enzootic transmission cycle coincided with a greater number of human cases with WNV-1 infection, as expected. However, a novel and interesting finding of this study, based on the comparison of the proportions of WNV-1 and WNV-2 infections in patients with WNND with those detected by screening of blood donors, was the demonstration that WNV-1 infection was associated with an increased risk of developing neuroinvasive disease than WNV-2 infection. The genetic basis and mechanism of increased neural tropism and damage are unknown and under investigation. It is worth noting that analysis of all the WNV-1 genomes generated so far from the outbreak for known pathogenic mutations^3^ identified the presence of a characteristic amino acid substitution in the non-structural protein NS3 (T249P), which has independently emerged in other epidemic strains of both WNV-1 and WNV-2^44^ and is associated with increased viremia and virulence in American crows.^45^ NS3-249P confers increased protein stability at high temperature,^9^ a trait that could be favourable during exceptionally hot weather conditions, like the summer 2022 in northern Italy.^46^ This mutation in the NS3 helicase/protease was associated with the NS3-H244Q mutation, which was found to be under positive selection in epidemic strains.^9^^,^^47^ Pairwise alignment of the Italian WNV-1 strain with the closest phylogenetically related WNV-Akela/France/2015 also identified the non-conservative mutation R15S in the N-terminal region of the NS4B protein. Although this mutation involves a poorly conserved residue of the NS4B protein among flaviviruses, it might have a role in viral biology and pathogenesis since this protein is involved in the formation of viral replication complex and in the counteraction of the host innate antiviral response.^48^ In particular, the N-terminal region of NS4B interacts with NS3 and promotes its helicase activity.^49^ Interestingly, the genetically related WNV-1 (Akela/France/2015) has circulated endemically in Southern France since 2000 with occasional spillovers to horses and humans.^50^ This epidemiological pattern changed abruptly in 2018, with the introduction of WNV-2, probably from northern Italy, which spread in Southern France, causing human and equine outbreaks.^50^

## Conclusions

In conclusion, a rapid expansion of the newly introduced WNV-1, co-circulating with the endemic WNV-2, was observed in norther Italy in 2022, where a large human outbreak occurred. The new WNV-1 strain appeared to be associated with an increased risk of neuroinvasive disease in humans compared with WNV-2 infection. An expanded genomic surveillance and an in-depth characterization of the virus phenotype is ongoing to define if the high incidence of severe disease in humans is due to increased virulence or if it is simply the result of more intense transmission among birds than WNV-2.

## Data availability

The data underlying this article will be shared on reasonable request to the corresponding author. Sequencing data have been submitted to the GenBank database under accession numbers OP609701-OP609810 and OP609812-609815.

## Authors’ contributions

M.P., F.M., D.F., Federica.G., E.Q., I.M., A.F., A.V., A.S., S.R., E.D.M., S.S., V.L., Federico.G., S.G., V.V., M.M. and A.B. collected samples, performed experiments and analysed the data. L.B., G.F., L.F., F.R., P.M., C.T. and G.C. designed the work. L.B., F.M., D.F., F.G., I.M. and A.F. drafted the manuscript and G.C. substantially revised the manuscript. All authors read and approved the final manuscript.

## Funding

This work was supported by the Veneto Region and by the European Union’s Horizon 2020 research and innovation programme (VEO, grant number 874735).

## Conflict of interest

None declared.

## Supplementary Material

Supplementary_data_taac125
