# Correction to: Rapid spread of a new West Nile virus lineage 1 associated with increased risk of neuroinvasive disease during a large outbreak in Italy in 2022

**DOI:** 10.1093/jtm/taae047

**Published:** 2024-03-26

This is a correction to: Luisa Barzon, Monia Pacenti, Fabrizio Montarsi, Diletta Fornasiero, Federica Gobbo, Erika Quaranta, Isabella Monne, Alice Fusaro, Andrea Volpe, Alessandro Sinigaglia, Silvia Riccetti, Emanuela Dal Molin, Sorsha Satto, Vittoria Lisi, Federico Gobbi, Silvia Galante, Giuseppe Feltrin, Valerio Valeriano, Laura Favero, Francesca Russo, Matteo Mazzucato, Alessio Bortolami, Paolo Mulatti, Calogero Terregino, Gioia Capelli, Rapid spread of a new West Nile virus lineage 1 associated with increased risk of neuroinvasive disease during a large outbreak in Italy in 2022, *Journal of Travel Medicine*, 2022, taac125, https://doi.org/10.1093/jtm/taac125

In the originally published version of this article, a citation was misnumbered in the following sentence:

“Testing for other vector-borne viruses (Usutu virus, USUV; Toscana virus, tick-borne encephalitis virus, dengue virus, Zika virus and chikungunya virus) was included in the differential diagnosis, as reported.”

This has been corrected.

