# Supplementary material for: Rapid spread of a new West Nile virus lineage 1 associated with increased risk of neuroinvasive disease during a large outbreak in Italy in 2022
Source: J Travel Med. 2022 Nov 4;31(8):taac125. doi: 10.1093/jtm/taac125 (PMC11646088; doi:10.1093/jtm/taac125)
Supplement: Supplementary_data_taac125 [file supplementary_data_taac125.pdf]

Supplementary Data

Rapid spread of a new West Nile virus lineage 1 associated with increased risk of neuroinvasive disease during a large outbreak in northern Italy, 2022: One Health analysis

Luisa Barzon, MD[1,2], Monia Pacenti, PhD[2], Fabrizio Montarsi, PhD[3], Diletta Fornasiero, DVM[3], Federica Gobbo, DVM[3], Erika Quaranta, PhD[3], Isabella Monne, PhD[3], Alice Fusaro, PhD[3], Andrea Volpe, MD[1], Alessandro Sinigaglia, PhD[1], Silvia Riccetti, PhD[1], Emanuela Dal Molin, DSc[1], Sorsha Satto, DSc[2], Vittoria Lisi, DSc[2], Federico Gobbi, MD[4], Silvia Galante, MD[5], Giuseppe Feltrin, MD[6], Valerio Valeriano, MD[7], Laura Favero, PhD[8], Francesca Russo, MD[8], Matteo Mazzucato, HSDG[3], Alessio Bortolami, PhD[3], Paolo Mulatti, PhD[3], Calogero Terregino, PhD[3], Gioia Capelli, PhD[3]

[1] Department of Molecular Medicine, University of Padova, via A Gabelli 63, 35121 Padova, Italy
[2] Microbiology and Virology Unit, Padova University Hospital, via Giustiniani 2, 35128 Padova, Italy
[3] Istituto Zooprofilattico Sperimentale delle Venezie, Viale dell'Università, 10 - Legnaro (PD), Italy
[4] Department of Infectious-Tropical Diseases and Microbiology, IRCCS Sacro Cuore Don Calabria Hospital, Negrar di Valpolicella, Italy
[5] UOC Medicina Trasfusionale, ULSS 6 Sede di Camposampiero, Azienda ULSS6 Euganea, via Cosma, 1 - Camposampiero (PD), Italy
[6] Regional Transplant Centre, Azienda Ospedale Università di Padova, Via Giustiniani 2, 35128 Padova, Italy
[7] Dipartimento di Prevenzione - AULSS 6 Euganea; Servizio di Igiene e Sanità Pubblica, UOSD Epidemiologia e Ambiente, Via Ospedale Civile, 22, 35100 – Padova, Italy
[8] Direzione Prevenzione, Sicurezza Alimentare, Veterinaria, Regione Veneto, Dorsoduro, 3493 - Rio Novo – 30123 Venezia, Italy

Supplementary Table 1. Primers used to amplify the whole WNV-1 genome.

Oligonucleotide	Sequence 5'→ 3'
WNV1_1f	ATTAACACGGTGCGAGCTGT
WNV1_1r	ACTGCGAGAAACGTGAGAGC
WNV1_2f	GGGAAGGCTGTCCATCAAGT
WNV1_2r	CTCCTTTGGTGAGGGAGTGTC
WNV1_3f	TCCTGCCCTCAGTAGTTGGA
WNV1_3r	TAGCTGGTTGTCTGTCTGCG
WNV1_4f	CTGGGAGTCGCAACCTTCTT
WNV1_4r	TGCTTCCCTTAGCCTTTCCG
WNV1_5f	TGGCACCACGATGAGAACC
WNV1_5r	CGGGGTCTCCTCTAACCTCTA

Supplementary Table 2. List of wild bird species tested positive for WNV-1, WNV-2, USUV, or co-infected with both WNV lineages.

**Common name**	**Latin name**	**WNV-1**	**WNV-2**	**WNV-1 + WNV-2**	**USUV**
Atlantic Canary	*Serinus canaria*		+		
Barn Owl	*Tyto alba*	+			
Barn Swallow	*Hirundo rustica*	+			
Common Blackbird	*Turdus merula*	+	+		+
Common Kestrel	*Falco tinnunculus*	+	+	+	
Common Pheasant	*Phasianus colchicus*	+			
Common Swift	*Apus apus*	+			
Common Woodpigeon	*Columba palumbus*	+	+	+	
Eurasian Jay	*Garrulus glandarius*	+	+	+	
Eurasian Magpie	*Pica pica*	+	+		+
Eurasian Scops Owl	*Otus scops*	+	+		
Eurasian Sparrowhawk	*Accipiter nisus*	+			
European Green Woodpecker	*Picus viridis*	+			
European Turtle Dove	*Streptopelia turtur*	+			
Hooded Crow	*Corvus cornix*	+	+		
House Sparrow	*Passer domesticus*	+			
Little Egret	*Egretta garzetta*	+			
Little Owl	*Athene noctua*	+	+	+	
Long-Eared Owl	*Asio otus*		+		
Mallard	*Anas platyrhynchos*				+
Purple Heron	*Ardea purpurea*		+		
Rook	*Corvus frugilegus*	+			
Tawny owl	*Strix aluco*				+
Western Cattle Egret	*Bubulcus ibis*	+			+
Yellow-Legged Gull	*Larus michahellis*	+	+		

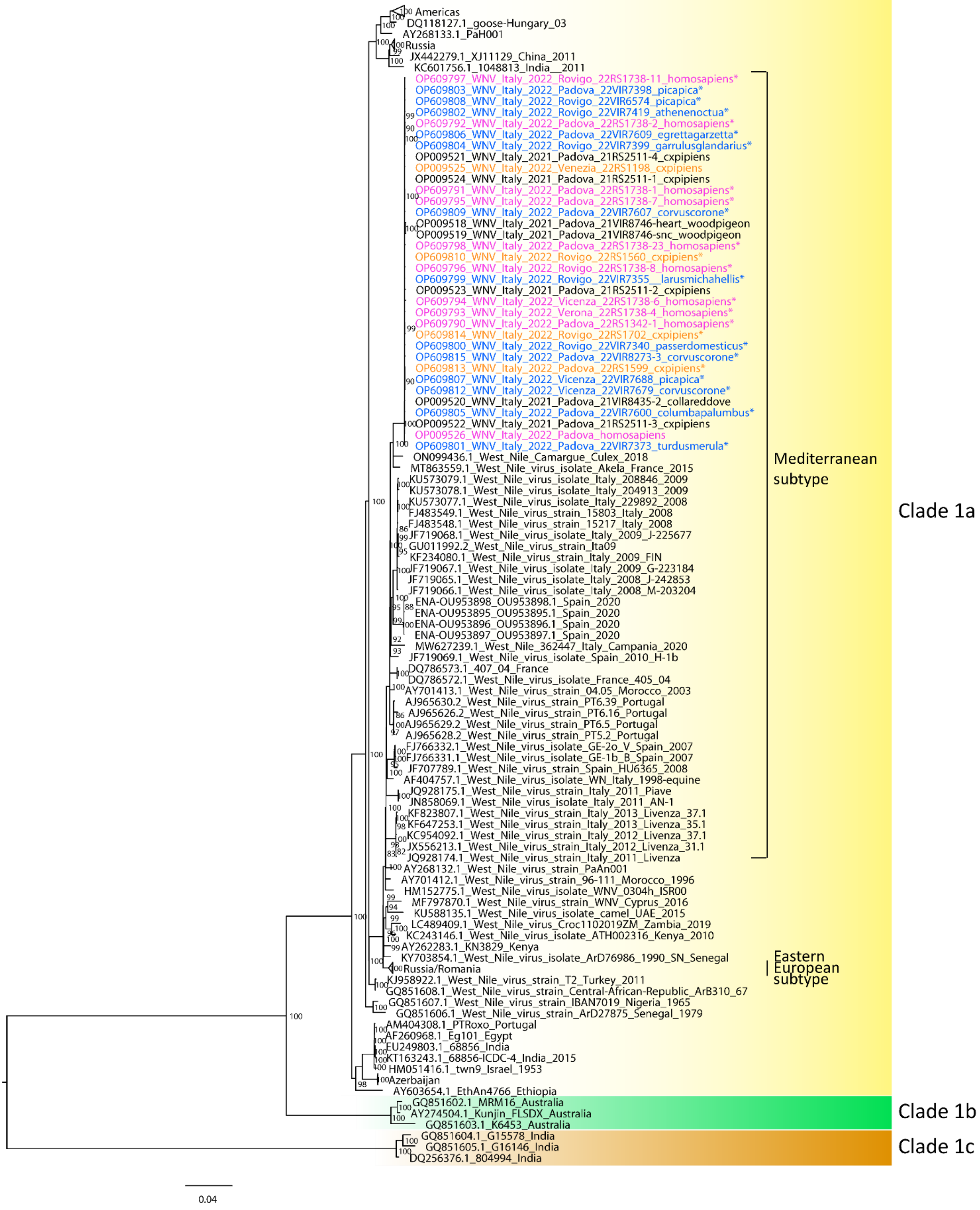

Supplementary Figure 1. Phylogenetic analysis of West Nile Virus lineage 1. The maximum-likelihood phylogenetic tree was constructed with IQ-TREE (GTR+F+I+G4) and 1,000 bootstrap replicates. Ultrafast bootstrap supports higher than 80 are indicated next to the nodes. Viruses collected in 2022 are identified by different colours according to the species of origin (fuchsia = humans; blue = wild birds; orange = mosquito pools). The viruses analysed in this study are marked with an asterisk. Background shading distinguishes three clades (clade 1a in yellow, clade 1b in green and clade 1c in brown).
